# Hair follicle mesenchymal stem cell exosomal lncRNA H19 inhibited NLRP3 pyroptosis to promote diabetic mouse skin wound healing

**DOI:** 10.18632/aging.204513

**Published:** 2023-02-14

**Authors:** Hongliang Yang, Yan Zhang, Zhenwu Du, Tengfei Wu, Chun Yang

**Affiliations:** 1 Department of Cardiology, China-Japan Union Hospital of Jilin University, Changchun 130031, China; 2 School of Public Health, Beihua University, Jilin 132033, China; 3 College of Basic Medicine, Beihua University, Jilin 132033, China; 4 Department of Laboratory Animal Science, China Medical University, Shenyang 110122, China

**Keywords:** HFMSCs, exosomes, lncRNA H19, diabetes, skin wound healing

## Abstract

Skin wounds caused by diabetes are a major medical problem. Mesenchymal stem cell-derived exosomes hold promise to quicken wound healing due to their ability to transfer certain molecules to target cells, including mRNAs, microRNAs, lncRNAs, and proteins. Nonetheless, the specific mechanisms underlying this impact are not elucidated. Therefore, this research aimed to investigate the effect of MSC-derived exosomes comprising long non-coding RNA (lncRNA) H19 on diabetic skin wound healing. Hair follicle mesenchymal stem cells (HF-MSCs) were effectively isolated and detected, and exosomes (Exo) were also isolated smoothly. Pretreatment with 30 mM glucose for 24 h (HG) could efficiently induce pyroptosis in HaCaT cells. Exosomal H19 enhanced HaCaT proliferation and migration and inhibited pyroptosis by reversing the stimulation of the NLRP3 inflammasome. Injection of exosomes overexpressing lncRNA H19 to diabetic skin wound promoted sustained skin wound healing, whereas sh-H19 exosomes did not have this effect. In conclusion, Exosomes overexpressing H19 promoted HaCaT proliferation, migration and suppressed pyroptosis both *in vitro* and *in vivo*. Therefore, HFMSC-derived exosomes that overexpress H19 may be included in strategies for healing diabetic skin wounds.

## INTRODUCTION

Chronic wounds that take a long time to heal owing to a variety of pathological reasons lead to financial and social problems as well as psychological stress [1]. With the aging of the population, the incidence of chronic wounds increases sharply owing to numerous age-associated illnesses, including diabetes. Chronic diabetic wounds are among the deadliest diabetic-related complications, affecting 15 percent of diabetic patients and contributing to a greater risk of amputation [2]. Under conditions of high glucose, inflammatory cells, fibroblasts, epidermal cells, and endothelial cells are dysfunctional, thus causing a delay in the wound healing process [3]. At present, it is still challenging to develop therapeutic strategies to improve wound healing in individuals with diabetes.

Mesenchymal stem cells (MSCs), including umbilical cord, bone marrow, and hair follicle stem cells, have aroused extensive research interest in the realm of regenerative medicine [4]. However, the mechanisms through which stem cells play a therapeutic role in the process of transplantation are still not well understood. Currently, a large amount of data confirm that transplanted stem cells play a positive role in tissue repair through a paracrine mechanism [5]. Among them, the cellular components called exosomes, produced during the paracrine process by stem cells, have attracted extensive attention. Exosomes are nanovesicles, and their biggest advantage is that they have almost no immunogenicity compared with the source cells themselves [6]. They are cup-shaped, with a density of approximately 1.13-1.19 g/ml and a diameter of approximately 40–100 nm. They contain lipid envelope and cytoplasmic components derived from cells. They are rich in mRNAs, microRNAs, lncRNAs, and proteins [7]. At present, they have been studied in a variety of disease models and have been shown to have certain effects, including renal damage repair enhancement, myocardial infarction area decrease, immunological response modulation, and skin wound healing. Stem cell-derived exosomes are expected to become a new promising direction of stem cell therapy [8].

Long non-coding RNAs (lncRNAs) have recently been demonstrated to exist in exosomes. It has been reported that exosomal lncRNAs can affect many diseases, such as diabetes, renal injury, and chronic skin wounds [9]. This suggests that lncRNAs may be transferred to cells through exosomes in the process of cell-to-cell communication and modulate gene expression [10]. We discovered a high expression of lncRNA H19 in hair follicle mesenchymal stem cell-derived exosomes (HF-MSCs-Exo). The lncRNA H19 has been depicted to perform a significant function in fibroblasts, enhancing proliferation and migration, and preventing apoptosis [11]. In this research, we also discovered that HF-MSCs-Exo can boost diabetic skin wound healing. However, the specific mechanism of exosomes in skin repair remains unclear. Excessive cell inflammation is a cause of chronic wounds [12]. Pyroptosis has recently been recognized as a type of programmed cell death. The activation of caspases, including Caspase-1, is mediated by inflammatory corpuscles, resulting in shear and polymerization of Gasdermin family members, including GSDMD, resulting in cell perforation and cell death [13]. Research reports have found that high glucose (HG)-mediated priming signal-induced NLRP3 (Nod-like receptor family pyrin domain comprising 3 mRNA) expression is reduced by exosomes from stem cells, indicating that exosomes perform instrumental functions in the modulation of NLRP3 gene expression of skin relative cells [14].

In this research, we postulated that lncRNA H19 delivered via HF-MSCs-Exo can regulate HG-induced human fibroblast (HDF) pyroptosis and enhance diabetic mouse skin wound healing. This research proposes a new mechanism through which HF-MSC-Exo promotes chronic skin wound repair, indicating that lncRNA H19 and exosomes could be a therapeutic strategy for diabetes focal skin defects.

## RESULTS

### Isolation of exosomes from HF-MSCs

**Figure 1:**
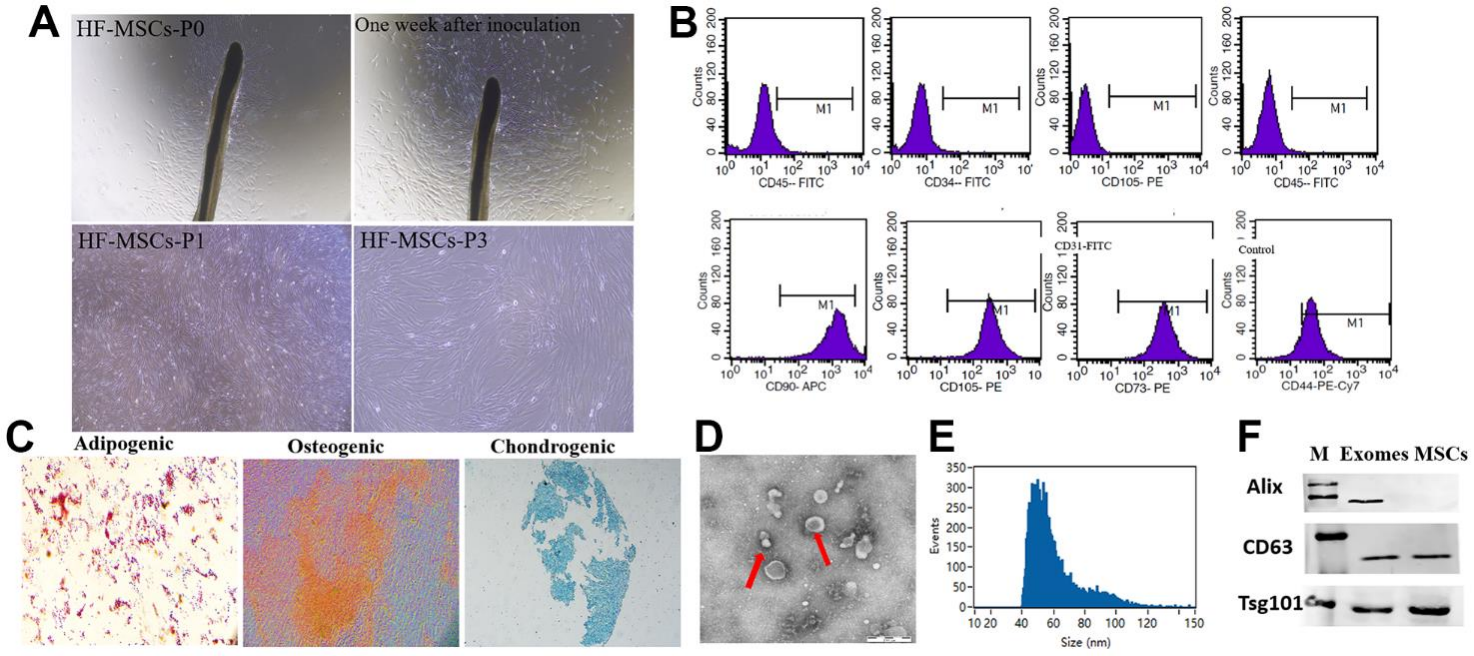
**Classification of hair follicle mesenchymal stem cells (HF-MSCs) and HFMSC-derived exosomes (HF-MSC-Exo).** (**A**) Morphological observation of MSCs (200×). (**B**) MSCs surface marker molecules identified by flow cytometry. HF-MSCs were positive for CD90, CD105, CD73, CD44 and negative for CD34, CD45 and CD31. (**C**) Cell lineage-induced chondrogenic, osteogenic, and adipogenic differentiation were evaluated by toluidine blue staining, alizarin red staining, and Oil-Red-O staining. Scale bar=100 μm. (**D**) Micrographs of transmission electron microscopy of purified HF-MSC-Exo, showing a spheroid shape. Scale bar=50 nm. (**E**) The size distribution of the HF-MSC-s was assessed utilizing dynamic light scattering. (**F**) Western blotting results indicated the positive expression of Alix, CD63, and Tsg101 protein in HF-MSC-Exo.

Fibroblast-like cells tended to migrate outward from the HFs ten days following the onset of HF culture. In tissue culture plates, HF-MSCs had a polygon-like and elongated shape. (Figure 1A). Flow cytometry assays with immunofluorescence staining revealed that fibroblast-like cells presented MSC surface markers (negative for CD34, CD31, and CD45 and positive for CD44, CD90, CD73, CD105) (Figure 1B). In adipogenic differentiation culture conditions, the cells illustrated the formation of lipid droplets in the cytoplasm after Oil Red O staining. Under an osteogenic growth environment, the cells’ shape transformed from fibroblast-like to osteoblast-like, and they demonstrated significant levels of alkaline phosphatase activity. Alcian blue staining revealed the presence of glycosaminoglycan on the 21st day of chondrogenesis, indicating the differentiation ability into chondrogenesis (Figure 1C). Therefore, hair follicle-derived fibroblast-like cells exhibit mesenchymal stem cell surface markers and displayed trilineage differentiation potential toward osteoblasts, adipocytes and chondrogenesis. Consequently, these cells were labeled as HF-MSCs.

Exosomes were isolated by ultracentrifugation from HF-MSC cells using 2% (w/v) exosome-deprived FBS. TEM illustrated that HF-MSCs produced vesicles with a distinct cup-shaped morphology (Figure 1D). According to nanoparticle tracking analyses, the particle size distribution of the vesicles was about 85 percent, ranging between 20 and 200 nm (Figure 1E). Western blotting revealed the existence of the exosomal marker proteins Alix, CD63, and Tsg101 (Figure 1F).

### Establishment of chronic hyperglycemic cell model

**Figure 2:**
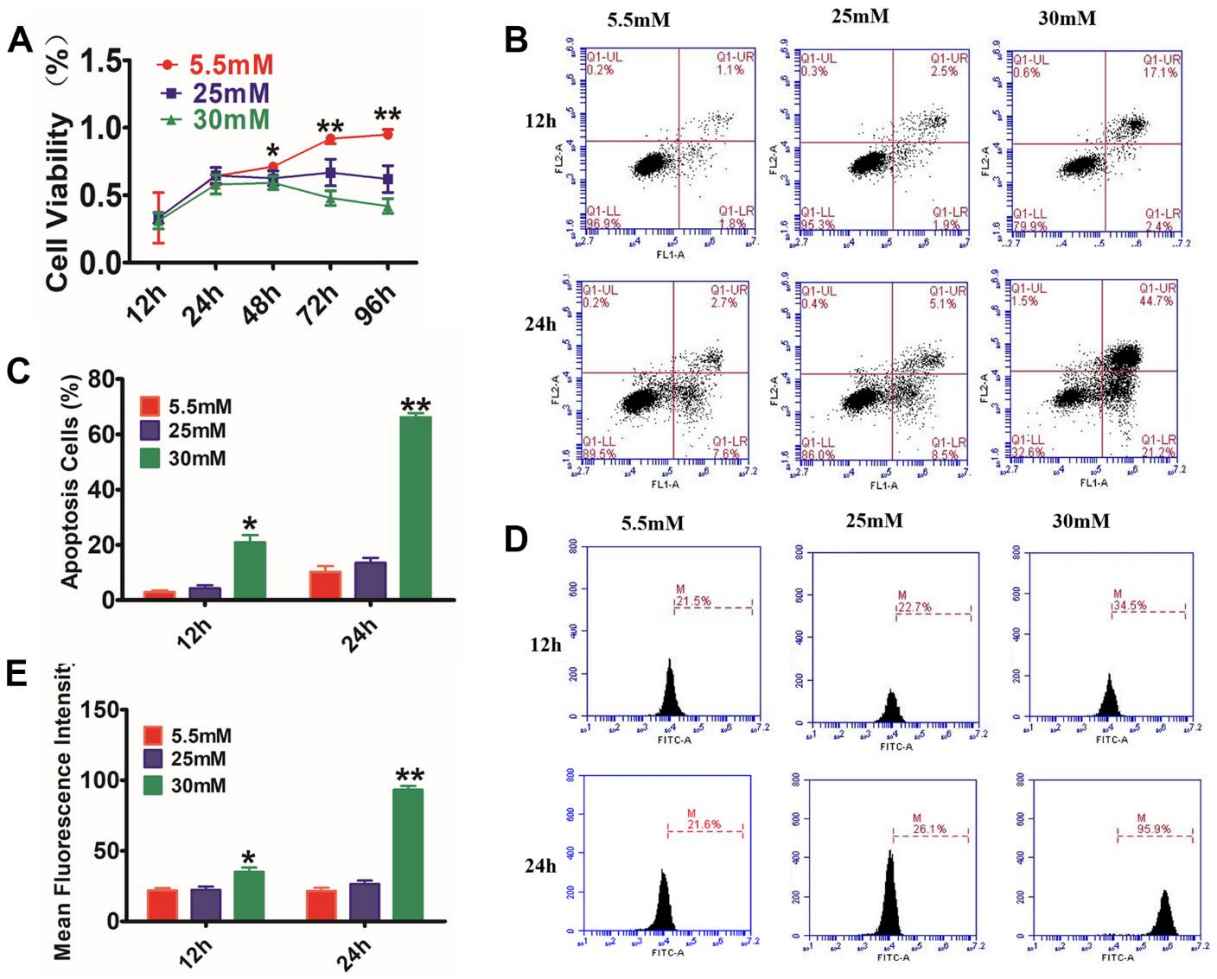
**Glucose effects on HaCaT cells by CCK-8 assay, AnexinV-PI, and ROS staining.** (**A**) HaCaT cells were treated with different concentrations of glucose for varying time periods, and then cell viability was measured with CCK-8. The data showed that glucose inhibited the cell viability of HaCaT cells in a dosage- and time-dependent way. (**B**) The annexin V-PI flow cytometry assay was utilized for the detection of the apoptosis rate of HaCaT cells, which were treated with 5.5, 25, or 30 mM glucose at different times. (**C**) The histogram results show that cell apoptosis increased following incubation with glucose. (**D**, **E**) Analysis of intracellular ROS levels using the flow cytometry assay. The histogram results show that the fluorescence intensity was increased following incubation with glucose. Control to 5.5 mM glucose, *P<0.05, **P<0.01, all results are representative of three separate experiments (means ± SD).

To determine the mechanism of the effects of exosomes on diabetic wounds, HaCaT cells were used to make the model chronic. HaCaT cells were treated with LG (5.5 mM) or HG (25 or 30 mM)12h to 96h. The CCK8 assay (Figure 2A) showed that HG stimulation resulted in a remarkable decrease in cell viability. Annexin V-PI staining in combination with flow cytometry assay (Figure 2B, 2C) illustrated that the proportion of PI-positive cells and annexin V-positive cells increased with time and concentration, being considerably higher compared with that in the control cohort (P<0.01). The ROS detection (Figure 2D, 2E) showed that the fluorescence intensity of ROS dramatically increased as early as 12 h after treatment with HG, being substantially higher compared with that of the control cohort (P<0.05); it continuously increased with time, reaching a maximum at 24 h.

### HG induced HaCaT cell injury by NLRP3 inflammasome-mediated pyroptosis

**Figure 3:**
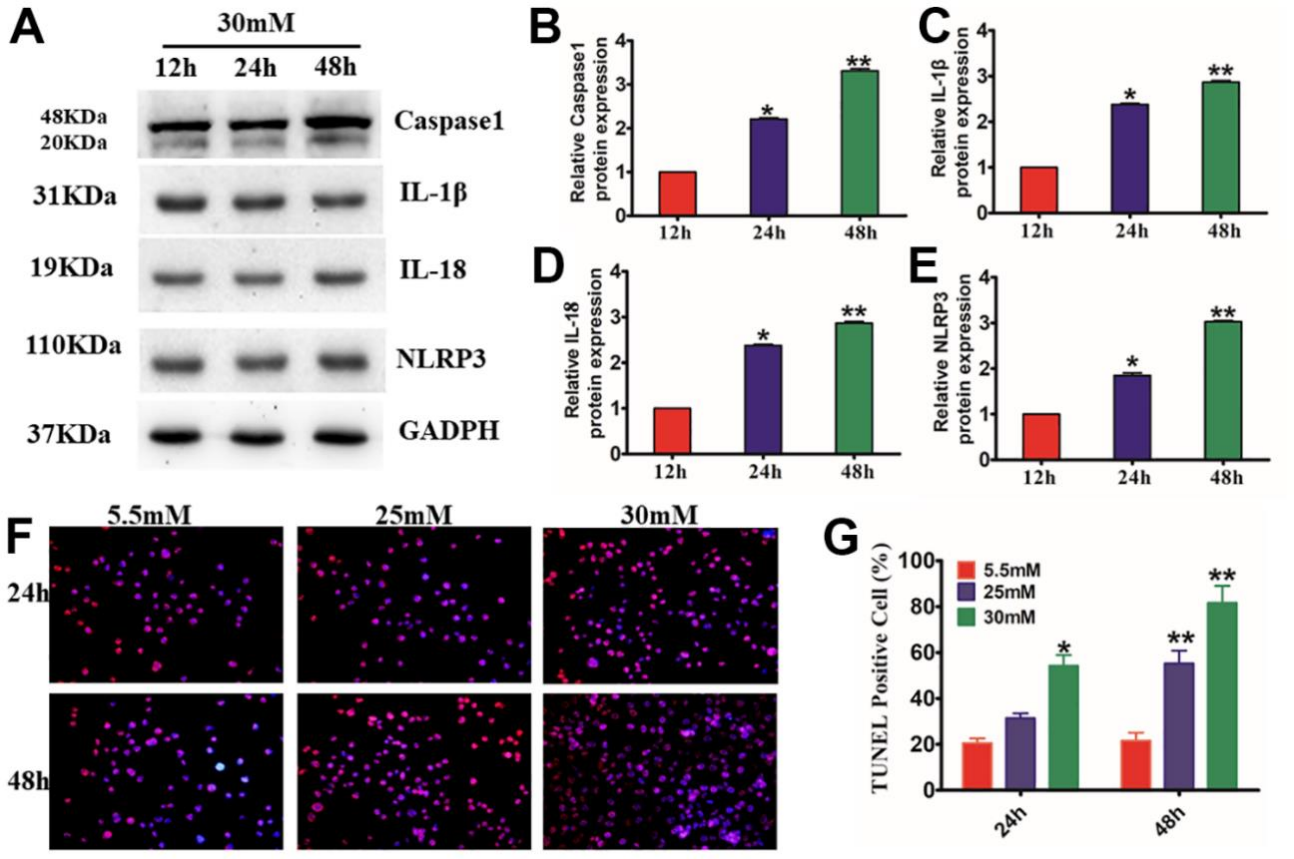
**Effects of the activation of NLRP3 inflammasome and expression of pyroptosis-related proteins in HG-induced HaCaT cells.** Western blotting (**A**), for caspase-1 (**B**), IL-1β (**C**), IL-18 (**D**), and NLRP3 (**E**) in HaCaT cells. (**F**, **G**), HaCaT apoptosis identified by the TUNEL assay (200×). *P<0.05 and **P<0.01versus the 5.5 mM cohort. Data are articulated as the mean±SD (n=6), and the experiment was redone independently three times.

The ASC, NLRP3, and caspase-1 multiprotein complex NLRP3 inflammasome are widely recognized as the primary pathway of inflammation and pyroptosis following cell damage. To examine the impact of Exo on the activation of NLRP3 inflammasomes *in vivo*, we first needed to study the injury model produced by treating HaCaT cells with HG. We found that HG treatment enhanced the activation of caspase-1 and the production of IL-18 and IL-1β. The increased reactivity of HaCaT cells to the inflammasome was supported by NLRP3 inflammasomes (P<0.01) (Figure 3A–3E). TUNEL staining results showed that HG-induced apoptosis increased in a time- and concentration-dependent way (P<0.01) (Figure 3F, 3G). These findings strongly suggest that HG stimulates IL-1β production and caspase-1 activation regulated by the NLRP3 inflammasome in HaCaT cells.

### HF-MSC-Exo remarkably attenuated HG-induced HaCaT cell death

**Figure 4:**
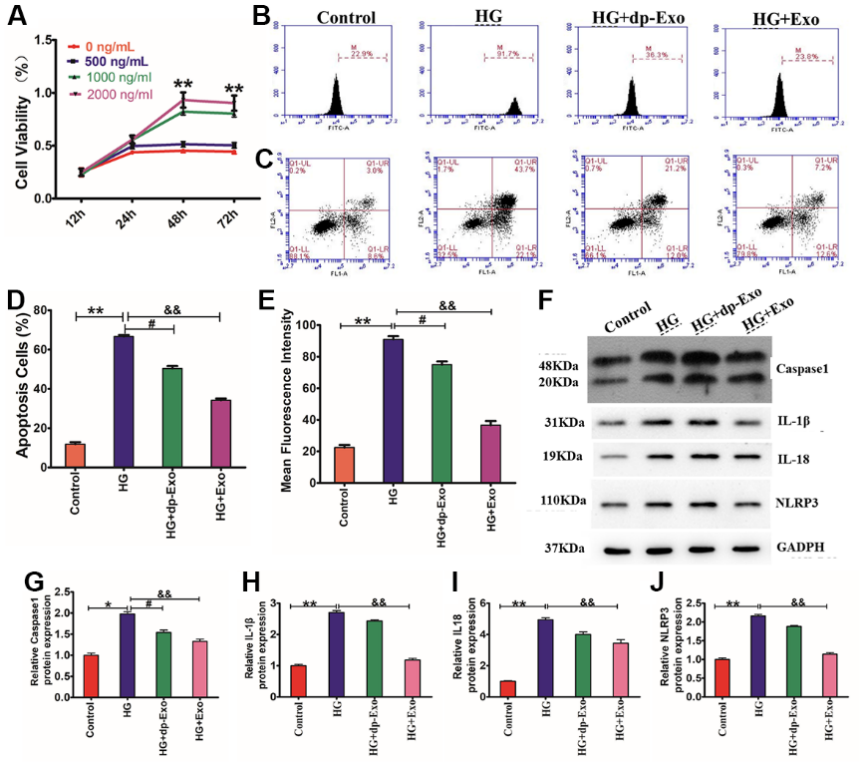
**HF-MSC-Exo inhibited HG-induced pyroptosis of HaCaT cells and promoted their proliferation.** (**A**) HaCaT cells were treated with HG and then treated with HFMSC-dp-Exo or HF-MSC-Exo for 24-72 h. The CCK-8 assay findings illustrated that the cell viability was higher in the presence of HF-MSC-Exo than in the control and also higher than the HF-MSC-dp-Exo cohort. (**B**, **C**) The flow cytometric assay results showed that the HF-MSC-Exo can inhibit HG-induced apoptosis of HaCaT cells. (**D**, **E**) The flow cytometric assay results showed that the HF-MSC-Exo can reduce HG-induced fluorescence intensity in HaCaT cells. Western blotting (**F**) for caspase-1 (**G**), IL-1β (**H**), IL-18 (**I**), and NLRP3 (**J**) in HaCaT cells. Compared to control: *P<0.05 and **P<0.01 representing three separate experiments (means ± SD).

**Figure 5:**
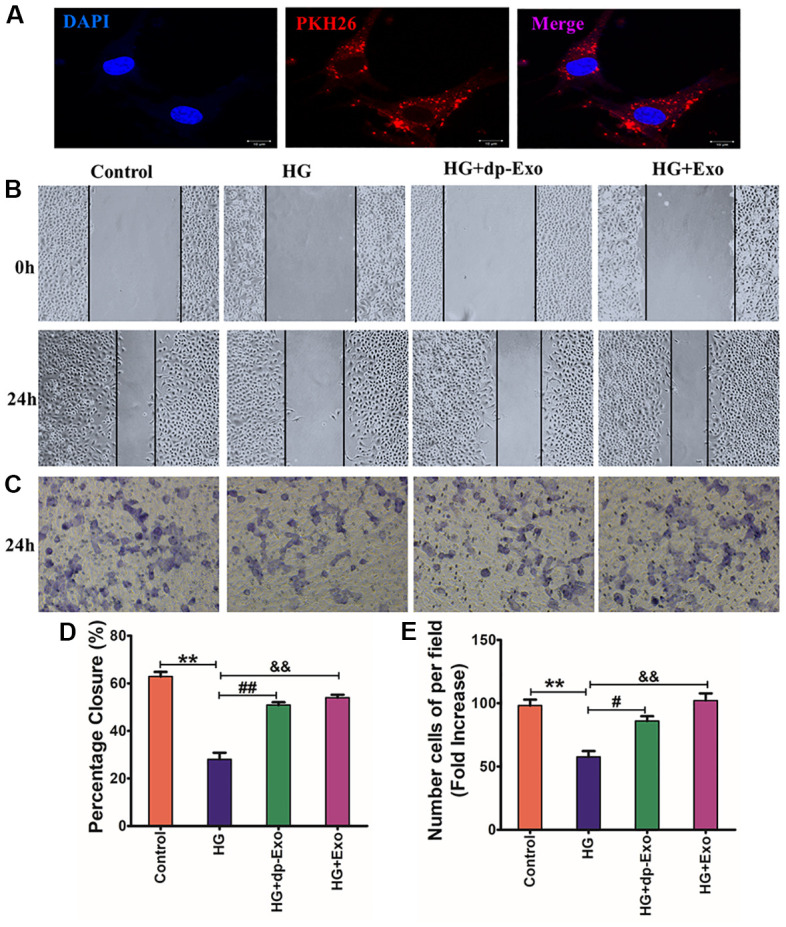
**HF-MSC-Exo can be uptaken by HaCaT cells and promote the migration of HG-treated HaCaT cells.** (**A**) HF-MSC-Exo was labeled with PKH26, a lipid membrane-intercalating dye. The labeled exosomes were introduced to the culture medium of HaCaT cells. After 24 hours, HaCaT cells were fixed, counterstained with Hoechst 33342, and analyzed by fluorescent microscopy. The images showed that the labeled HF-MSC-Exo entered into the cytoplasm of HaCaT cells. Scale bar=200 μm. HaCaT cells were treated with HG and subsequently cultured in the presence of HF-MSC-Exo or HF-MSC-dp-Exo for 24 hours. The control cohort was the normal HaCaT cells. (**B**, **C**) HaCaT cells skin wound healing and cell migration experiment after treated with different culture medium for 24hours. (**D**, **E**) Quantified the proportion of wounded area closure and cell migration rate. (n=3); **P<0.01 Control vs HG; ^##^*P*<0.01, dp+Exo vs HG; ^&&^*P*<0.01, Exo vs HG). Scale bar = 200 μm. All results are representative of three independent experiments (means ± SD).

Increasing research evidence indicates that paracrine signaling performs an instrumental function in stem cell-based therapy. To investigate the underlying mechanism through which paracrine signaling enhances cutaneous healing of wounds, we made HaCaT cells exposures to H_2_O_2_ and then to different concentrations of HFMSCs-Exo for various periods of time. CCK8 assay illustrated that the HaCaT cells cultivated in the presence of HFMSCs-Exo had considerably greater viability as opposed to that of the control cohort (0 ng/ml). Furthermore, the viability of HaCaT cells cultivated in HF-MSCs-Exo exhibited an increase in the dosage range from 500 ng/ml to 2000 ng/ml, and from 12-48 h (Figure 4A). Furthermore, we compared the effect of HF-MSCs-Exo on HG-induced increase in the levels of ROS and apoptotic rate of HaCaT cells with that of HFMSCs-dp-Exo (GW4869) ROS flow cytometry results showed that the mean fluorescence intensity of the cells was reduced more than HG cohort in the HF-MSC-Exo cohort (Figure 4B, 4D). Similar to ROS measurement, the percentage of apoptotic cells in the HF-MSCs-Exo cohort was considerably lesser than that in the HG cohort (Figure 5C, 5E). However, neither the percentage of apoptotic cells nor the intensity of ROS fluorescence showed any significant difference between the HG and HF-MSCs-dp-Exo cohorts (P>0.05). We found that HF-MSC-Exo treatment reduced the activation of caspase-1 and the release of IL-18 and IL-1β. The reduction in the inflammasomes of HaCaT cells was supported by NLRP3 inflammasomes (P<0.01) (Figure 4F–4J). These data clearly indicate that HF-MSC-Exo inhibited IL-1β, IL- 18 production, and activation of caspase-1 and reduced the NLRP3 inflammasome in HaCaT cells.

### HF-MSC-Exo could transfer to HaCaT cells and promote HaCaT cell migration

To detect the internalization of HF-MSC-Exo by HaCaT cells, PHK26 fluorescent dye was used to label HF-MSC-Exo. Exosomes labeled with PKH26 were subjected to incubation with HaCaT cells. In a duration of 24 h, the fluorescence was observed in the cytoplasm and nucleus of the cells under the fluorescence microscope, suggesting that the HFMSC-Exo could be taken up by HaCaT cells and distributed intracellularly into the cytoplasm and nucleus (Figure 5A).

Keratinocyte migration has been known to be a crucial phase in the healing of cutaneous wounds. HaCaT cells were pretreated with HG, followed by incubation with HF-MSC-dp-Exo or HF-MSC-Exo for 24 hours to investigate the impact of HF-MSC-Exo on cellular migration. The cell scratch assay illustrated that the healing rate of wound areas was larger in the HF-MSC-Exo cohort contrasted with that in the HF-MSC-dp-Ex cohort (P<0.05) (Figure 5B, 5C). Transwell assay indicated that incubation with HF-MSC-Exo dramatically increased the number of migrated cells relative to the HFMSC-dp-Exo cohort (P<0.05) (Figure 5D, 5E).

### Construction of cell lines with stable lncRNA H19 expression

**Figure 6:**
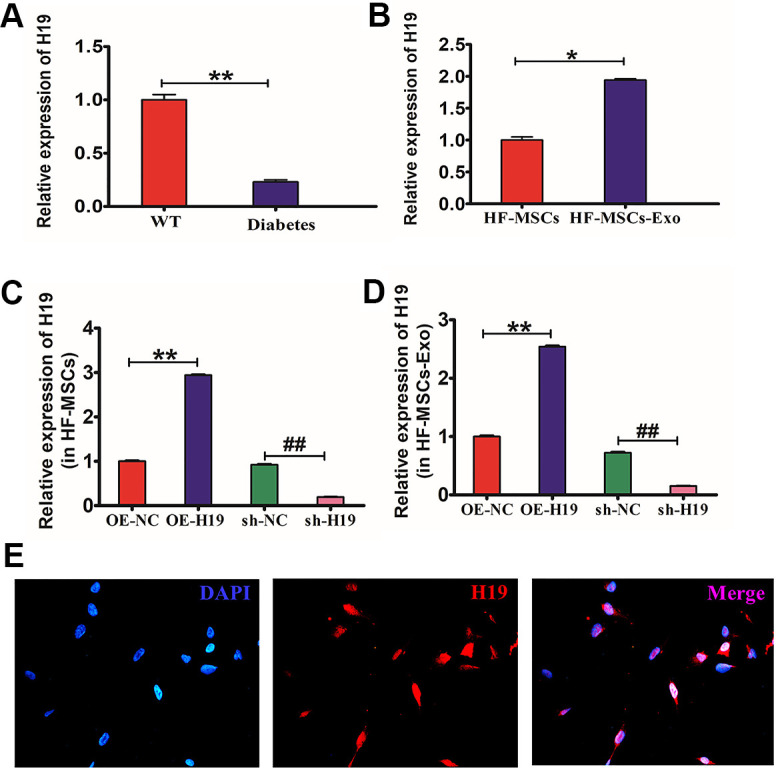
**Production HF-MSCs and HF-MSC-Exo with lncRNA H19.** (**A**) qRT-PCR was utilized to identify the lncRNA H19 expression in mouse diabetic skin. *P<0.05 WT vs Diabetes. (**B**) qRT-PCR was utilized to identify the lncRNA H19 expression in HF-MSCs and HF-MSC-Exo. *P<0.05 MSC vs MSC-Exo. (**C**, **D**) qRT-PCR was employed to ascertain the efficiency of overexpressing or silencing lncRNA H19 in HF-MSCs and HF-MSC-Exo.**P<0.01, OE-H19 vs OE-NC ^##^*P*<0.01, sh-H19 vs sh-NC. (**E**) Subcellular localization of lncRNA H19 in HFMSCs detected by FISH (400×). Data measured are articulated as mean ± SD.

Existing research evidence has indicated that the lncRNA H19 may serve as a ceRNA to modulate miRNAs expression to regulate skin wound healing and glioma. Using qRT-PCR, we found that the levels of lncRNA H19 expression were low in the diabetic skin compared to the normal skin (Figure 6A). While it is highly expressed in HF-MSC-Ex (Figure 6B), the effects of lncRNA H19 on the biological characteristics of HG-treated HaCaT cells were examined by overexpressing or silencing lncRNA H19 expression. The efficiency of overexpressing or silencing lncRNA H19 in HFMSCs cells met the requirements for further experiments (P<0.05) (Figure 6C). Exosomes derived from the transfected cells also showed overexpression or downregulation of lncRNA H19 expression. (Figure 6D). RNA- FISH result illustrated that lncRNA H19 was present in the nucleus and cytoplasm of HFMSCs. (Figure 6E).

### HF-MSC-Exo carrying of lncRNA H19 regulates HaCaT proliferation, migration, and apoptosis via the NLRP3 pyroptosis signaling pathway

**Figure 7:**
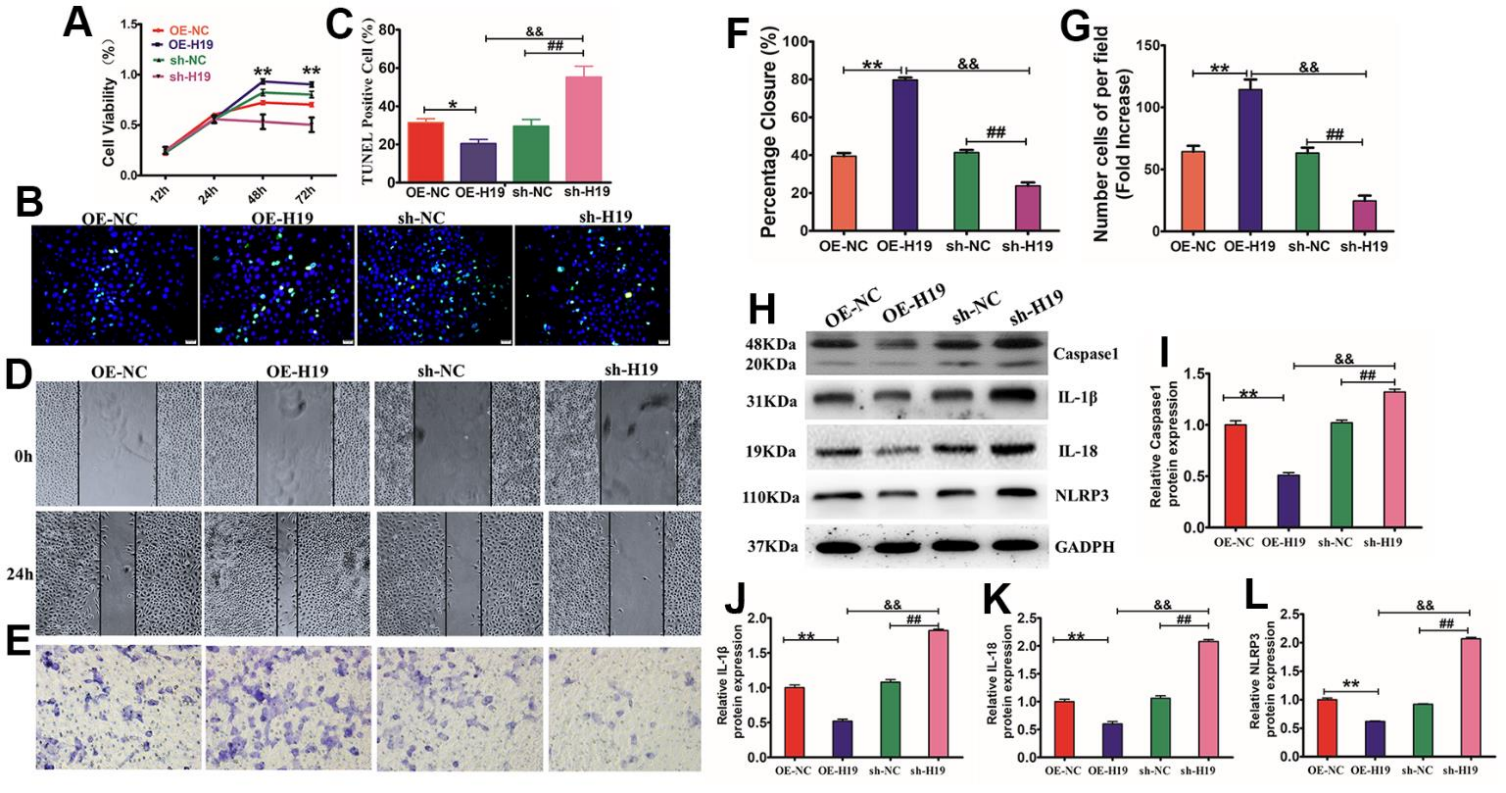
**Exosomes overexpressing lncRNA H19 affected HaCaT cell proliferation, apoptosis, migration, and pyroptosis.** (**A**) HaCaT cells were treated with HG and subsequently cultured in the presence of OE-H19-exosomes, sh-H19-exosomes, and NC-exosomes. The CCK-8 assay findings illustrated that cell viability was higher in the OE-H19-exosomes cohort than in the sh-H19-exosomes cohort. (**B**, **C**) HaCaT apoptosis identified by TUNEL assay (200×). *P<0.05 and **P<0.01versus the 5.5 mM cohort. Data are articulated as mean±SD (n=6), and the experiment was redone separately three times. (**D**, **E**) HaCaT cells were treated with OE-H19-exosomes, sh-H19-exosomes, and NC-Exo were exposed to a wound-healing assay and transwell migration assay for 12 hours. Scale bar =200μm. (**F**, **G**) Statistic the wound area closure and the number cell of per filed. Scale bar=200 μm. All results are representative of three separate experiments (means ± SD). Western blotting (**H**) for caspase-1 (**I**), IL-1β (**J**), IL-18 (**K**), and NLRP3 (**L**) expression in HaCaT cells. **P<0.01 OE-19 *vs* NC; ^##^*P*<0.01, sh-19 *vs* NC; ^&&^*P*<0.01, OE-19 *vs* sh-19), representative of three independent experiments (means ± SD).

Considering that HF-MSCs-Exo can deliver lncRNA H19 to HaCaT cells through exosomes, the modulatory function of exosomes in proliferation, migration, and apoptosis of HaCaT cells was investigated by incubating HaCaT cells with normal HF-MSCs-Exo (NC) or HF-MSCs-Exo overexpressing H19 (OE-H19) or having downregulated levels H19 (sh-H19). The results showed that OE-H19 delivery promoted HaCaT cell proliferation and migration by suppressing apoptosis, while sh-H19 had the reverse effect (P<0.01), implying that HF-MSC-Exo carrying of lncRNA H19 regulates fibroblast proliferation, migration, and apoptosis (Figure 7A–7G). NLRP3 is considered to be an essential inhibitor of the pyroptosis signaling pathway, which performs a critical function in regulating cell growth, differentiation, proliferation, and apoptosis. Treatment of HG-induced cells with different exosomes showed that OE-Exo inhibited the activation of caspase-1 and GSDMD cleavage. Therefore, lncRNA H19 is also essential for the modulation of the activation of NLRP3 inflammasome and cleavage of GSDMD in HaCaT cells (Figure 7H–7L). In summary, these findings illustrate that the anti-inflammatory benefit of lncRNA H19 in exosomes from HF-MSCs may be an important gene for skin wound healing.

### HF-MSC-Exo carrying of lncRNA H19 mediates pyroptosis to promote wound healing in diabetes mice

**Figure 8:**
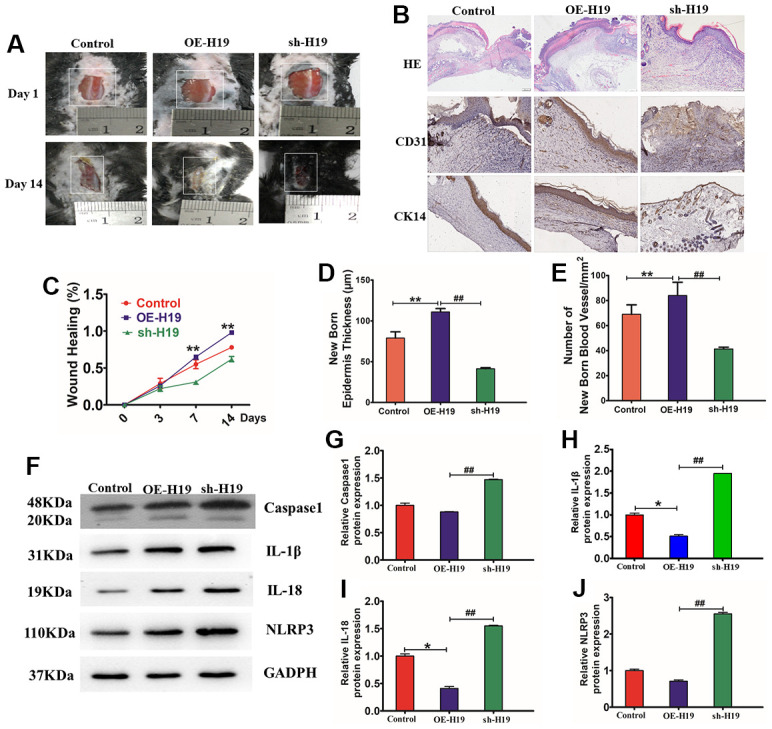
**HF-MSC-Exo carrying lncRNA H19 promote mouse skin wound healing.** (**A**) Representative images displaying mouse skin wound healing. (**B**) Wound histology after H&E staining. Tissue sections acquired from the wound site on day 14 after different injections were stained with antibodies against cytokeratin 14 and CD31. Scale bar = 200 μm. (**C**) Statistic the wound healing percent. (**D**) Quantitative analysis of the thickness of the new epidermis. (**E**) Quantitative analysis of the number of blood vessels. n = 3 per cohort. **P<0.01 OE-H19 contrasted with Control, ##P<0.01 OE-H19 compared with sh-H19. (**F**) Protein bond diagram of caspase-1 (**I**), IL-1β (**J**), IL-18 (**K**), and NLRP3 ascertained by western blot analysis. (**G**–**J**) Relative protein expression of caspase-1, IL-1β, IL-18, and NLRP3 normalized to GAPDH was evaluated by western blot analysis.

To examine the impact of HF-MSC-Exo in the process of wound repair in diabetic mice, we constructed a diabetic mouse skin wound model and injected the tissues surrounding the wound with HF-MSC-Exo overexpressing H19 (OE-H19) or with downregulated H19 (sh-H19), followed by immunohistochemical and H&E staining to observe wound healing. Exosomes overexpression of H19 (OE-H19) significantly accelerated the wound healing process *in vivo*, as confirmed by the presence of thicker granulation tissues and fewer inflammatory cells surrounding the wound (Figure 8A–8E). Western blot analysis of the skin wound tissue revealed that treatment with HF-MSC-Exo OE-H19 led to a lower level of caspase1, IL-1β, and TNF-α, illustrating that HF-MSC-Exo overexpressing H19 can mitigate the NLRP3 inflammation of diabetic skin wounds in mice. Therefore, HF-MSC-Exo carrying of overexpressing the lncRNA H19 quicken the process of wound healing in diabetes skin. OE-H19 exosomes protected against HG-induced injury in HaCaT cells by suppressing NLRP3 inflammasome-mediated pyroptosis by inhibiting the protein expression of IL-18, IL-1β, Caspase-1, and NLRP3 P<0.01 (Figure 8F–8J).

## DISCUSSION

Wound healing is known to be an intricate and highly regulated process that is key to maintaining the function of the skin barrier [15]. Patients with diabetes mellitus suffer from slow or even nonunion, which can lead to diabetic foot and amputation [16]. It is the main complication causing a high disability rate among patients with diabetes and can threaten their lives in severe cases [17]. The healing of surface wounds requires the synergy of many factors to restore the barrier function of injured skin [18]. The diabetic wound is a complex condition that is characterized by oxidative stress, long-term chronic inflammation, neovascularization, peripheral neuropathy, extracellular matrix accumulation, and remodeling imbalance [19]. It is critical to research the fundamental processes of skin wound healing so as to create viable treatments. lncRNAs have been regarded as conserved, tissue-specific, and endogenous molecules that regulate a wide range of biological processes via the adsorption of miRNAs [20].

lncRNA H19 is a highly conserved gene that has gained extensive research attention in the fields of cancer and cardiovascular disease [21, 22]. Furthermore, it has recently been linked to the skin and smooth muscles, stem cell differentiation and embryonic development [23, 24]. lncRNA H19 has been found to be expressed in abundance in embryonic tissues, demonstrating its prospective role in shaping the growth of embryonic cells [25]. By conducting a series of *in vivo* and *in vitro* experimentations, Chang et al. have discovered the existence of MSC-derived exosomal [9]. Downmodulation of lncRNA H19 has also been strongly linked to the occurrence of dysplasia and the progression of hip dislocation [26]. Notably, long non-coding RNAs (lncRNAs) have been found in exosomes, indicating that lncRNAs could be transferred from one cell to another via exosomes in the process of cell-to-cell contact and additionally modulate gene expression in host cells [27, 28]. In this research, we investigated the mechanism that underlie HF-MSC-derived exosomal lncRNA H19 and determined by virtue of a blend of *in vivo* and *in vitro* experimentations that it functions in the NLRP3 pyroptosis signaling pathway. Depending on the available data, it is reliable to infer that HF-MSC-Exo carrying of high levels of lncRNA H19 may stimulate the process of wound healing in mice with DFU.

As an important accessory organ of the skin, hair follicles not only have a series of basic physiological functions but also play an important role in repairing skin damage during the period of skin damage. Hair follicle stem cells are abundant in sources and can only be obtained by hair extraction. The main types of stem cells obtained from hair follicles are melanocytes, mesenchymal stem cells, and keratin stem cells. These cells are located in the specific microenvironment of hair follicles, and their unique periodic activities are regulated by multiple signaling pathways [29]. *In vivo*, treatment of wounds with HF-MSC resulted in shorter wound length and faster fibroblasts differentiation to myofibroblasts in the wound, which contributed to a shortened proliferation stage [30]. Exosomes, which have good therapeutic characteristics, have been found in MSC-conditioned media. Exosomes have received a lot of interest due to their paracrine signaling characteristics and a new form of noncellular treatment [31, 32]. MSCs secreted exosomes (MSCs-Exo) have been demonstrated to have protective effects against liver fibrosis *in vivo* and also modify epithelial to mesenchymal transition (EMT) markers by elevating the count of E-cadherin-positive cells while decreasing the count of N-cadherin- and vimentin-positive following MSCs-Ex transplantation in mice [33, 34]. In this research, we effectively harvested exosomes from HF-MSCs, and we demonstrated that HF-MSC-Exo can enhance HG-induced HaCaT proliferation, migration and inhibit apoptosis. However, the underlying mechanism is not clear.

Numerous research reports have illustrated that a decline in the inflammatory response contributes to a delay in the repair rate of skin wounds in chronic wound healing models [35]. Pyroptosis occurs simultaneously with an inflammatory reaction, where inflammasomes, gasdermin D, pro-inflammatory cytokines (IL-18, and IL-1β), and caspase-1 could be seen [8]. We demonstrated that HaCaT cells can be induced to undergo pyroptosis with caspase-1, NLPR3, ILβ, and IL18. NALP3 signaling is necessary for the natural process of wound healing. A number of pro-inflammatory cytokines, such as TNF-α, IL-1β, and IL-6, play a key role in the healing of skin wounds. It has been reported that NALP3 signaling is crucial for the optimum healing of skin wounds [36, 37]. The inflammasomes activation via the topical injection of extracellular ATP considerably enhanced skin wound healing by upregulating the inflammatory response in the initial wound healing phase. Topical ATP delivery might contribute to the development of novel, successful therapies for speeding skin wound healing [36]. In our study, HF-MSC-Exo significantly enhanced HaCaT cell proliferation, migration and inhibited apoptosis. The results showed that Exo can inhibit NLRP3 related proteins including caspase-1, ILβ, and IL18. A prior study found that H19 had anti-inflammatory properties [38] and illustrated that H19 was inhibited in sepsis patients and that the H19 overexpression might have a reversal effect on the LPS-induced myocardial dysfunction and production of anti-inflammatory cytokines both *in vitro* and *in vivo*. H19 has been reported to perform a function in modulating mitochondrial apoptosis in the process of cardiac ischemia-reperfusion injury via the miR-877-3p/Bcl-2 axis [39]. Therefore, it is apparent that H19 has a protective function in skin wounds. However, whether H19 can mitigate skin wounds in diabetic mice by inhibiting pyroptosis in chronic skin wound healing requires further evidence. Next, we found that lncRNAH19 was more highly expressed in diabetic mouse skin than in normal mouse skin. We overexpressed lncRNA H19 in HF-MSCs and harvested the exosomes, OE-H19-Exo, which were found to enhance HaCaT proliferation, migration, and inhibit apoptosis following high expression of the inflammation factor NLRP3, caspase-1, ILβ, and IL18 in the model of the injury cell model.

In summation, these reasonable findings enabled us to postulate a molecular mechanism that underlie DFU therapy by which HF-MSC-released exosomal lncRNA H19 suppresses inflammation, enhances proliferation and migration, and suppresses apoptosis of fibroblasts, thus ameliorating the damage of DFU and speeding up the process of wound healing. To conclude, HF-MSC-Exo lncRNA H19 might serve as an auspicious treatment strategy for DFU. However, the present study is still in the preclinical stage, and more research is necessary to investigate the mechanism of action.

## CONCLUSIONS

Our study revealed a significant role of HF-MSC-Exo that overexpress lncRNA H19 in the development of strategies against diabetic skin wounds. HF-MSC-Exo overexpressing lncRNA H19 promoted HaCaT proliferation, migration, and pyroptosis suppression both *in vitro* and *in vivo*.

## MATERIALS AND METHODS

### Isolation, culture, and osteogenic, adipogenic, and chondrogenic differentiation of HF-MSCs

HF-MSC isolation was performed as previously reported [40]. Succinctly, a minimum of 20 hairs with intact follicles was manually plucked from the occipital area of volunteers’ scalp. The hair was rinsed three times in phosphate-buffered saline (PBS; Life Technologies, Carlsbad, CA, USA) comprising 1 percent penicillin/streptomycin solution (P/S; 100 IU/ml penicillin, 100 IU/ml streptomycin; HyClone, Victoria, Australia). Following the cutting of the hair shafts, the hair follicles were seeded at the base of a 24-well plate (Corning, Tewksbury, MA, USA), with each well containing one piece of hair. They were then subjected to culturing in 100 ml of Dulbecco’s modified Eagle medium: nutrient mixture F-12 (DMEM/F12; Life Technologies) comprising 10 percent fetal bovine serum (FBS; HyClone), 1 percent P/S, and 2 ng/ml basic fibroblast growth factor (bFGF; Life Technologies) and subsequently subjected to incubation at 37° C over the night in a humidified environment comprising 5% CO_2_. Each well received 400 μl of culture media the following day, and the medium was replaced after every 3 days. When proliferating HF-MSCs achieved 80% confluence, sub-culturing was performed HF-MSCs were employed for experimentations at passages 3–8.

The expression of embryonic stem cell (ESC) markers and MSC surface markers was evaluated utilizing immune fluorescence, as described earlier [41]. Concisely, fixing of HF-MSCs was done using 4 percent paraformaldehyde for 15 minutes at a temperature of 25° C, followed by blocking using 1 percent bovine serum albumin (BSA; Roche Diagnostics, France), and incubation using primary mouse anti-human antibodies against CD31, CD105, CD90, CD34, (eBioscience, San Diego, CA, USA), AIF (Cell Signaling Technology, Danvers, MA, USA) at a dilution ratio of 1:200 at a temperature of 4° C over the night. After washing thrice with PBS, HF-MSCs were subjected to incubation with Alexa Fluor 488/555-conjugated anti-mouse secondary antibodies (dilution ratio of 1:400; Cell Signaling Technology) at 25° C for 60 minutes in darkness. HF-MSCs were then rinsed thrice in PBS, followed by counterstaining with Hoechst 33342 (dilution ratio of 1:10,000; Life Technologies) at a temperature of 25° C for five minutes in darkness. Subsequently, fluorescence microscopy (Olympus, Japan) was utilized to visualize the cells’ images. To perform flow cytometry analysis, HF-MSCs were obtained via centrifugation and subsequently treated using similar methods applied in immunofluorescence staining before being evaluated utilizing a FACS Calibur flow cytometer (BD Biosciences, San Jose, CA, USA). Cell Quest Software (BD Biosciences) was used to analyze the data.

Osteogenic and adipogenic differentiation of HF-MSCs was conducted as earlier reported [42]. Succinctly, culturing of the cells was done in either adipogenic differentiation medium comprising of high-glucose DMEM (H-DMEM, Life Technologies) comprising 10 percent FBS, 1 mM dexamethasone, 0.5 mM isobutyl-methylxanthine (Sigma-Aldrich, St. Louis, MO, USA), 10 mM insulin (Sigma-Aldrich), and 200 mM indomethacin (Sigma-Aldrich). Oil Red O staining (Sigma-Aldrich) was utilized two weeks following adipogenic stimulation to detect intracellular lipid droplets.

With regards to osteogenic differentiation assays, culturing of HF-MSCs was done in H-DMEM comprising 10 percent FBS, 0.1 mM, and 10 nM β-glycerophosphate (Sigma-Aldrich) for four weeks. Alizarin red S staining (Sigma-Aldrich) was carried out at the latest stage of the culturing to assess the development of calcium nodules.

With respect to chondrogenic differentiation, HF-MSCs spheres were produced by hanging drop culture using 20 μL of 8 × 10^6^ cells/mL; spheres were then subjected to culturing in chondrogenic-induction medium comprising of (D)MEM, 10 percent FBS, 6.25 μg/mL insulin, 50nM of ascorbate-2-phosphate (Sigma-Aldrich), and 10 ng/mL transforming growth factor-beta 1 (PeproTech, London, UK). After every three days, the culture media were changed. Cartilages were identified three weeks following chondrogenic stimulation utilizing toluidine blue staining (Dingguo, Beijing, China) as per the guidelines stipulated by the manufacturer.

### Isolation and identification of MSC-Exo

The exosomes were isolated from MSCs as earlier reported [43]. Succinctly, HF-MSCs at passage 4 were cultured in DMEM comprising 10 ng ml−1 bFGF and 10 percent (w/v) FBS deprived of exosomes by centrifugation at 120,000×g over the night at a temperature of 4° C. The HF-MScs were cultivated in DMEM with 2 percent (w/v) exosome-FBS for 24 hours after achieving 80 percent confluence. To pellet the cells, the culture media were obtained and centrifugated at 300×g for 10 minutes at a temperature of 4° C. Next, the supernatant was extracted and centrifugated at 16,500×g (Optima™ L-100XP ultracentrifuge; Beckman Coulter, Palo Alto, CA, USA) at a temperature of 4° C for 20 minutes, and filtered using a 0.22-μm filter to get rid of cell debris. This medium was set as the conditioned medium (HF-MSCs-CM). The filtrate was centrifugated at 120,000×g at a temperature of 4° C for 90 minutes. The exosomes were extracted and assigned as HF-MSC-derived exosomes (HFMSCs-Exo). HFMSCs-Exo were resuspended in PBS and kept at a temperature of −80° C. A bovine calf albumin (BCA) kit (Beyotime, Shanghai, China) was used to determine the protein content in the HF-MSCs-Exo. Nanoparticle tracking analysis was adopted to assess the concentration and size distribution of exosomes utilizing ZetaView particle tracker from Particle Metrix (Germany), where each of the Nanoparticle Tracking Analysis (NTA) assessments for the various procedures for each participant was done in triplicate. Transmission electron microscopy (TEM; FEI Tecnai 12; Philips, Amsterdam, The Netherlands) was utilized to examine the morphology of HF-MSCs-Exo. Western blotting was employed in evaluating the expression levels of TSG101 (1:500, ProteinTech, Chicago, IL, USA), Alix (1:1,000, Abcam, Cambridge, MA, USA), and CD63 (1:500, Millipore, Temecula, CA, USA), in exosomes. The exosome-free medium was assigned as exosome-deprived HF-MSCs conditioned media with exosomes inhibitor GW4689 (HF-MSCs-dp-Ex).

### RNA-FISH

The subcellular localization of lncRNA H19 in HaCaT cells (Immortalized human epidermal cells) was detected using fluorescence *in situ* hybridization (FISH) techniques, following the guidelines described in the Ribo lncRNA FISH Probe Mix (red) kit (lnc10000; Ribobio, Guangzhou, Guangdong, China). Coverslips were put onto the wells of a 24-well plate, and cells were grown at a concentration of 6 × 10^4^ cells in each well. Upon achieving approximately 80% confluence, the cells were fixed using 1 ml 4 percent paraformaldehyde followed by treatment with 2 mg/ml acetylation reagent, glycine, and proteinase K. The cells were subsequently prehybridized for 1 hour at a temperature of 42° C using a 250-ml prehybridization solution followed by hybridization over the night at 42° C using a 250-ml hybridization solution comprising 300 ng/ml probe lncRNA H19. Staining of the nucleus was done for five minutes using DAPI diluted by PBS comprising Tween 20 at 1:800. After using an anti-fluorescence-quenching agent to mount the cells, they were examined utilizing a microscope and then photographed.

### Cell proliferation assay

The proliferation of HaCaT cells was identified utilizing Cell Counting Kit-8 (CCK-8; Dojindo Molecular Technologies, Tokyo, Japan) [44]. Succinctly, 2 × 10^3^ cells were placed in triplicate in 96-well plates and subsequently cultured in L-DMEM medium supplemented with (25, 30) μM glucose (Sigma-Aldrich) and 2% (w/v) FBS, or different concentrations of exosomes (0, 500, 1,000, 2,000 ng/ml). After 12, 24, 48, 72, or 96 h, each well received CCK-8 reagent and plates were further incubated for an extra 1 h. After the incubation approached completion, a microplate reader (Synergy H1; Biotek, USA) was employed to determine the absorbance of each well’s supernatant at 450 nm. The outcomes are articulated as the mean ± standard deviation from 3 separate experiments.

### Apoptosis assays and ROS detection

Annexin V-PI Apoptosis Detection Kit (Sangene, Tianjin, China) and a ROS assay kit (EMD Millipore, Billerica, MA, USA) were utilized to perform apoptosis analysis as per the protocols provided by the manufacturer [45, 46]. Concisely, HaCaT cells were plated into 6-well or 96-well tissue culture plates (triplicate) at a concentration of 5 × 10^4^cells/cm^2,^ followed by culturing in DMEM comprising 10 percent FBS for 24 hours. After cultivation, aspiration of the culture medium was carried out, and the cells were rinsed in PBS and subjected to culturing in DMEM comprising 2 percent FBS in the presence or absence of (control) 30 mM glucose for an extra 12-72 h. At the latest stage of cultivation, propidium iodine-Annexin V staining (San Jian, Tianjin, China) and reactive oxygen species (ROS) staining (Millipore) were conducted to determine apoptosis and ROS generation, respectively, at the specified time points following the manufacturer’s guidelines. Flow cytometry (BD Biosciences) was subsequently used to analyze the cells. For the detection of ROS, the probe dilution was done to a final concentration of 10 μM using a serum-free medium. The probe was introduced to the cells, placed in an incubator at 37° C with 5% CO_2_ for 30 minutes, and then washed with a serum-free medium. Flow cytometry (BD Biosciences) was then employed to examine the cells.

### TUNEL assay for cell apoptosis

HaCaT cells induced by HG and then added into different mediums including exosomes overexpressing Lnc H19 or sh-H19 were added to the cell cultures. TUNEL staining was performed utilizing a TUNEL Apoptosis Assay Kit (MK500, TaKaRa Bio Inc, Shiga, Japan), as described previously [47]. TUNEL^+^ cells were manually quantified with an inverted fluorescence microscope (Carl Zeiss, Oberkochen, German).

### Protein extraction and Western blot

Extraction of total proteins from the wound edge tissue and HaCaT cells was done using Radio immunoprecipitation assay buffer (RIPA) buffer (Boster Biological Technology, China) as per the protocol stipulated by the manufacturer. The protein concentration was ascertained utilizing the BCA protein assay kit (Boster Biological Technology, China). Extracted proteins (100 μg) were isolated by a 10 percent SDS polyacrylamide gel (Bio-Rad, Hercules, CA, USA). Blocking of the membranes was done with 5 percent nonfat milk for 2 h at 25° C ensued by the incubation of the PVDF membrane with the antibodies over the night at 4° C. The rinse of the membranes was done thrice for 8 minutes each in moderate volume of 1 × TBST and then incubated with secondary antibody in a 37° C incubator for 2 hours. Protein visualization was done by an enhanced chemiluminescence system using a Fluor Chem FC system (Alpha Innotech, San Leandro, CA, USA). Image J densitometry analysis was conducted to analyze protein bands, and the fold expression is designated as the relative protein expression. Antibodies information is in Table 1.

**Table 1:** Antibodies information.

**Name of antibody**	**Antibody dilution**	**Company**	**Catalogue number**
Caspase1	1:1000	Cell signaling	4542s
NLRP3	1:1000	Cell signaling	9532s
IL-18	2.5 μg/ml	IBL	1C-205
IL-1β	1:1000	Cell signaling	2556s
GADPH	1:1000	Cell signaling	4280s
CD31	1:200	Santa Cruz	SC-133494
CK4	1:500	Santa Cruz	SC-1060
CD90	1:200	eBioscience	2154875
CD105	1:200	eBioscience	2205006
CD73	1:200	Invitrogen	2105949
CD44	1:200	eBioscience	2087672
CD31	1:200	eBioscience	2152408
CD45	1:200	eBioscience	31011A20
CD34	1:200	Cell signaling	35695

### Cell transfection

H19 overexpression plasmid (pcDNA3.1-H19) was purchased from Shanghai Gene Pharma, and H19 smart silencer was purchased from RiboBio (Shanghai, China). HF-MSCs (60%–70% confluency) were plated into six-well plates. Lipofectamine3000 transfection reagent (Thermo, USA) was employed to conduct cell transfection with the pcDNA3.1-H19 plasmid. The smart silencer was transfected utilizing Thermo’s Lipofectamine RNAiMAX transfection reagent (80 nM). The culture medium was changed with a new one 6 hours following incubation.

### Transwell assay and scratch assay

To examine cell migration, transwell and cell scratch tests were conducted [48]. Succinctly, HaCaT cells were plated in the upper chamber of a transwell (8-μm pore filters; Corning, Corning, NY, USA) as per the guidelines provided by the manufacturer. HaCaT cells were plated in 6-well plates at a seeding concentration of 5 × 10^4^ cm^2^, followed by culturing over the night in DMEM comprising 10 percent (w/v) FBS. In the day that followed, the cells were rinsed thrice in PBS before being cultured in 30 mM glucose DMEM comprising 2 percent (w/v) exosome-free FBS for 24 hours. A 200-μl micropipette tip was utilized to scratch HaCaT cells grown in the 6-well plates. The HaCaT cells cultivated in the 6-well plates and Transwell were rinsed three times in PBS before being cultured for 24hours in DMEM comprising 2 percent (w/v) exosome-deprived FBS, HF-MSCs-dp-Exo, or HF-MSCs-Exo in 6-well plates or 100 μl DMEM comprising 2 percent (w/v) exosome-deprived FBS in the upper Transwell chamber. In the lower Transwell chamber, 600 μl DMEM comprising 2 percent exosome-free FBS, HFMSC-dp-Exo, or 1,000 ng/ml HFMSCs-Exo were added. After the Transwells were taken away from the tissue culture plate, the culture medium in the upper chamber of the Transwell was aspirated ensued by fixing of the HaCaT cells in the Transwells using methanol, drying with a laminar flow hood, and staining with Hoechst 33342 (1:10,000, 25° C, 2 min; Life Technologies, Carlsbad, CA, USA). The HaCaT cells that were left in the upper Transwell chamber were extracted with a cotton tab and washed thrice in PBS. A fluorescent microscope (Olympus) was used to visualize the HaCaT cells that were remained on the lower surface of the top transwell chamber. ImageJ software (NIH, Bethesda, MD, USA) was used to examine HaCaT cells in five view areas chosen at random. Photographs were taken for HaCaT cells cultivated by 6-well plates. Migration under the above-mentioned culture conditions was measured. Computation of the migration rates was done as indicated below: (distance of cells at scratching time point − distance of cells 24 h post-scratching)/(distance of cells at scratching time point).

### RNA isolation and quantitation

TRIzol reagent (Invitrogen, Carlsbad, CA, USA) was utilized to obtain total RNA from HFMSCs, exosomes, and diabetes mouse skin or normal mouse skin as per the protocols stipulated by the manufacturer. The Prime Script RT Reagent Kit (Takara, Beijing, China) was employed for reverse transcription of RNA into cDNA. The amplification reactions were conducted using a qRT-PCR system (ABI 7500, Thermo Fisher Scientific, MA, USA) utilizing 1 μl cDNA and 1 μl primer (Sangon Biotech, Shanghai, China). A 20 μl reaction volume was employed, comprising amplification SYBR Premix Ex Taq kit (Takara) and primers. The reactions were done premised on the conditions illustrated below: denaturation at 95° C for 3 minutes, followed by 40 cycles of denaturation at 95° C for 3 seconds and 60° C for 30 seconds. All reactions were repeated in triplicates for each sample. The relative miRNA expression or mRNAs expression was assessed by the 2^-ΔΔCt^ method and normalized to glyceraldehyde 3-phosphate dehydrogenase (GAPDH), in that order. The primers are H19 (F:5’-TACAACCACTGCACTACCTG-3’, R:5’-TGGAATGCTTGAAGGCTGCT-3’), GAPDH (F: 5’-AATCCCATCACCATCTTCCA-3’, R: 5’-TGGACTCCACGACGTACTCA-3’).

### Diabetic model induction

All of the animal experiments were carried out in strict adherence to the standards approved by the Institutional Animal Care Committee and the China Association of Laboratory Animal Care. The male SPF C57 mice weighing between 20–25 g were procured from the Laboratory Animal Department of China Medical University and kept at the Institutional Animal Center of Jilin University, Jilin, China. A week was spent for mice to adapt to their new surroundings. After adaptation, they were initially fed with a high-fat diet for 8 weeks, followed by intraperitoneal injection (i.p.) with STZ solution (140 mg/kg, dissolved in 0.1 M sodium citrate buffer) [49, 50]. Following the injection, the mice were given an HF diet for an additional four weeks. The level of blood glucose (BG) was measured from the mice’s tail tip. Once the levels of BG greater than 11.2 mmol/L were achieved, the development of the diabetic mice model was judged effective.

### Full-thickness skin defects and diabetes mouse cutaneous wound healing

After the successful preparation of the diabetes model, surgical scissors were employed to create a full-thickness excisional wound measuring 0.8 cm × 0.8 cm on the dorsal skin of each mouse [51]. Three cohorts of mice, each comprising six animals, were grouped at random. Subcutaneous injections of 100 g OE-H19 100 L, 100 g sh-H19 100 L, or 100 g PBS were administered at the edges of each wound margin (PBS cohort). As pointed out earlier, the wounds were dressed in one oil gauze layer topped with three cotton gauze layers.

### Gross inspection, and immunofluorescence, and H&E staining

Images of the wound sites were obtained on days 7 and 14 following wounding for a thorough examination of wound healing. A transparent film was used to display the contour along the wound edge, and the wound closure rate was determined as indicated below: [(original wound area−new wound area)/original wound area] ×100. After sacrificing the mice using an overdose of anesthetic, we obtained skin specimens that included wounds and surrounding tissues. The specimens were subsequently fixed using 10 percent (w/v) buffered formaldehyde/PBS, embedded in paraffin, dissected at 5-μm thick slices in the middle of the wound, followed by staining with hematoxylin and eosin (H&E). Observation and photographing of the tissue sections were done with the aid of a microscope. The newly formed epidermis was designated as epidermal tissue delimited by hair follicle-free dermis. The computation of the histological wound healing rate was performed as illustrated below: (length of the newly formed epidermis)/(length of newly formed epidermis + non-healed epidermis).

In order to identify epidermal formation, dermal angiogenesis and scar tissue in injured skin, immunofluorescence staining was conducted. Concisely, skin slices were deparaffinized followed by rehydration, and eventual blocking in 1 percent (w/v) BSA/PBS at a temperature of 25° C for 30 minutes. The tissue sections were subjected to incubation over the night at a temperature of 4° C using rat anti-mouse primary antibodies against cytokeratin 14 (1: 100; Abcam) and CD31 (1:100; Cell Signaling Technology) and subsequently rinsed thrice with PBS. In order to track the nuclei, the sections were subjected to incubation for 30 minutes at 25° C with Alexa Fluor-488/555-conjugated anti-mouse secondary antibody, followed by counterstaining with Hoechst 33342 (Invitrogen). A fluorescent microscope equipped with a digital camera (Leica DFC500, Wetzlar, Germany) was used to examine and take pictures of the slices. Five fields in each tissue section were chosen at random and examined at a magnification of ×400. From each of the three mice in each cohort, three sections were selected. The averaged optical densities of the expression of CK14 and CD31 were determined utilizing Image-Pro Plus (Media Cybernetics, Rockville, MD, USA). At each time point, five fields chosen at random were inspected and utilized to compute the average optical density per unit area for each cohort.

### Statistical analysis

SPSS (version 17.0; IBM Corp., Armonk, NY, USA) was utilized to execute statistical analyses. Data are articulated as the mean ± standard deviation (SD) for ≥3 separate experiments. One-way ANOVA was utilized to compare multiple cohorts. The student’s t-test was utilized to compare paired cohorts. Statistical significance was adjusted at P<0.05.
